# Supplementary material for: Directed self-assembly of fluorescence responsive nanoparticles and their use for real-time surface and cellular imaging
Source: Nat Commun. 2017 Dec 1;8:1885. doi: 10.1038/s41467-017-02060-8 (PMC5709404; doi:10.1038/s41467-017-02060-8)
Supplement: Supplementary file 1 — Supplementary Information [file 41467_2017_2060_MOESM1_ESM.pdf]

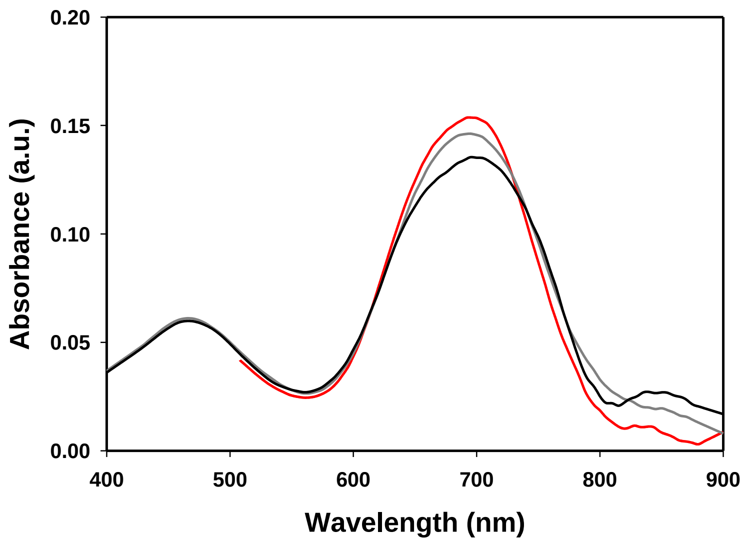

Supplementary Figure 1. Stability of DSA particles. Absorbance spectra of aqueous NP**1**-P_{188} solution (5 µM 1, 1.19mM P_{188}) recorded after one day (red), one week (grey) and two weeks.

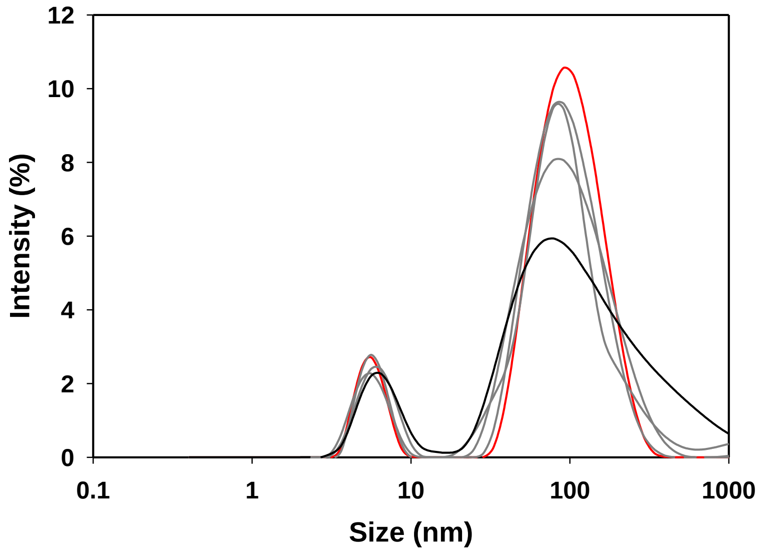

Supplementary Figure 2. Stability of DSA particles. DLS traces taken following four 1:1 serial dilutions starting from aqueous NP**1**-P_{188} solution of 5 µM **1**, 1.19mM P_{188} at 25 °C.

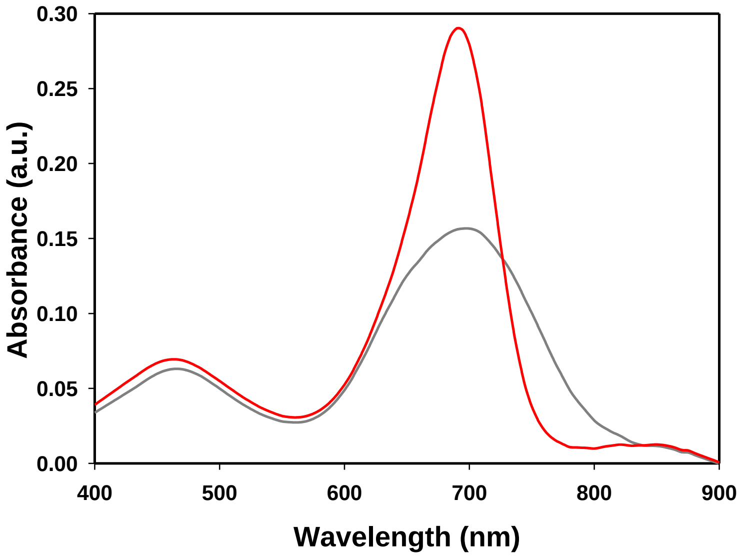

Supplementary Figure 3. Temperature effect on absorbance. Absorbance spectra of aqueous NP**1**-P_{188} (5 µM 1, 1.19mM P_{188}) at 25 °C (grey trace) and 65 °C (red trace).

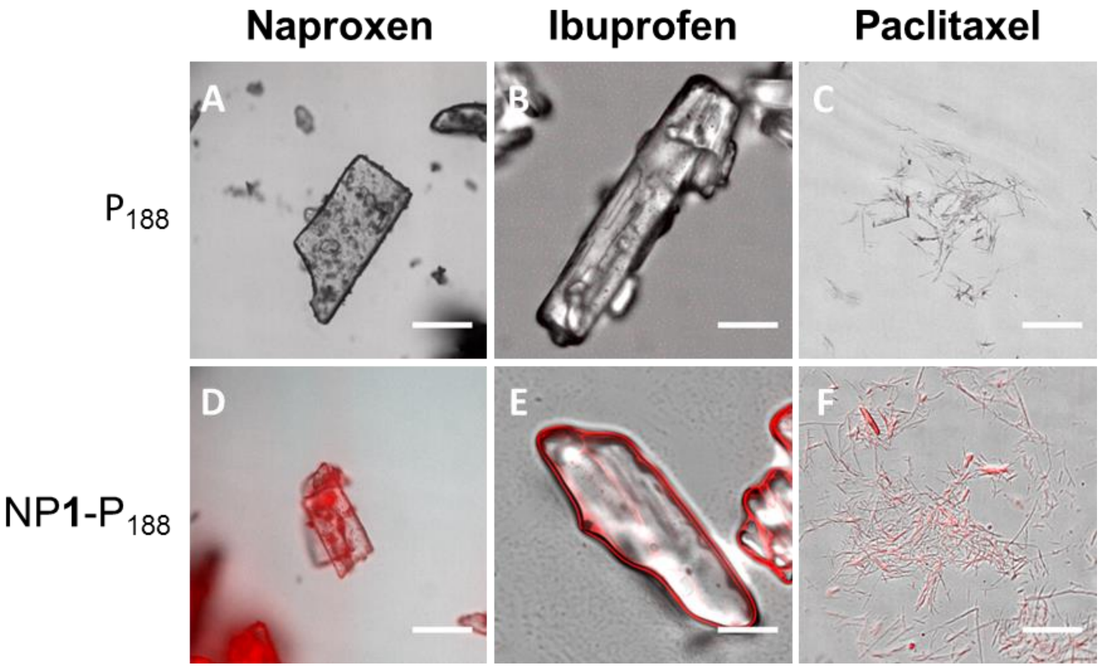

Supplementary Figure 4. Fluorescence and differential interference contrast (DIC) images of drug crystal following treatment with either P_{188} (control), or NP**1**P_{188} for 2 h Fluorescence (red) overlaid on and DIC crystal images of naproxen, ibuprofen and paclitaxel following treatment with 1.19 mM P_{188} alone (A-C) or NP**1**-P_{188} (D-F). Scale bars = 70 µm.

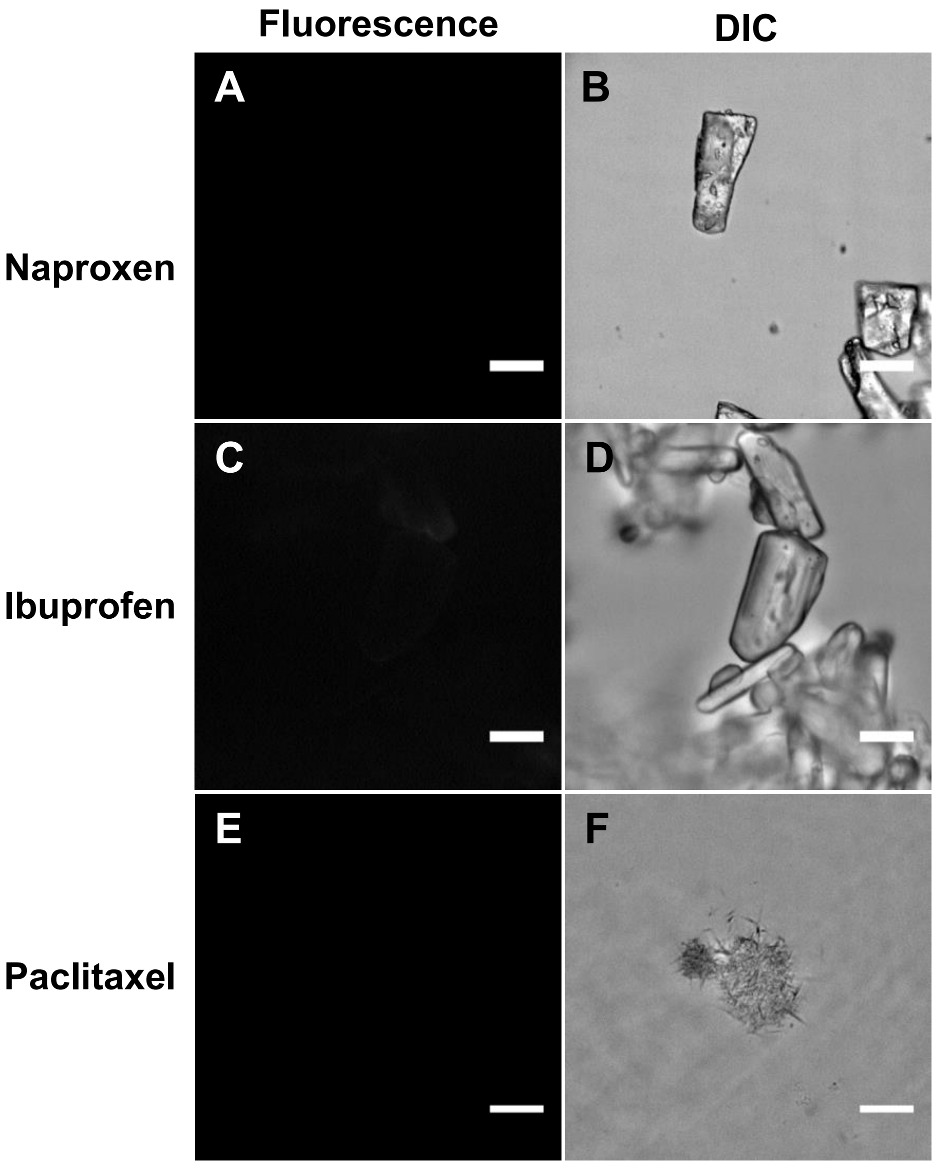

Supplementary Figure 5. Negative control crystal imaging experiments. Fluorescence and differential interference contrast (DIC) images of naproxen (A and B), ibuprofen (C and D) and paclitaxel (E and F) following treatment with 1.19 mM P_{188} alone for 40 min. Scale bar = 50 µm.

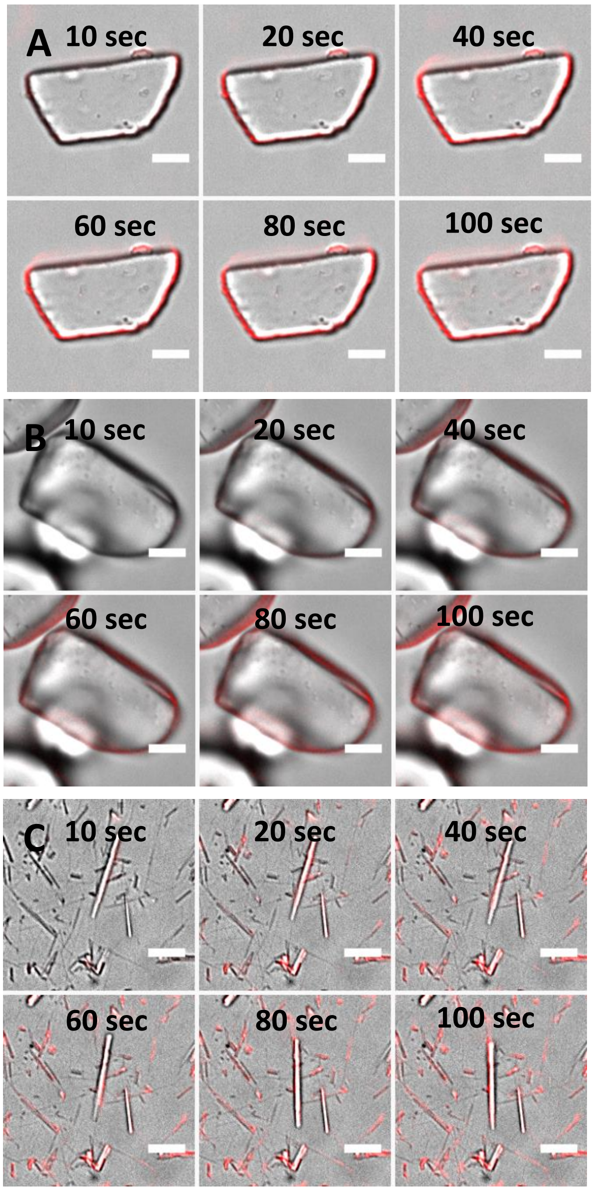

Supplementary Figure 6. Real-time fluorescence imaging of adsorption at drug crystal surfaces over 120 sec following treatment with aqueous NP**1**-P$_{188}$. Red coloured fluorescence overlaid on DIC images from Figure 13. Real-time fluorescence imaging of adsorption at drug crystal surfaces at time points of 10, 20, 40, 60, 80 and 100 sec following treatment with aqueous NP**1**-P$_{188}$. **A**. Naproxen (Supplementary Movie 4); **B**: Ibuprofen (Supplementary Movie 5); **C**: Paclitaxel (Supplementary Movie 6). Scale bars = 10 μm.

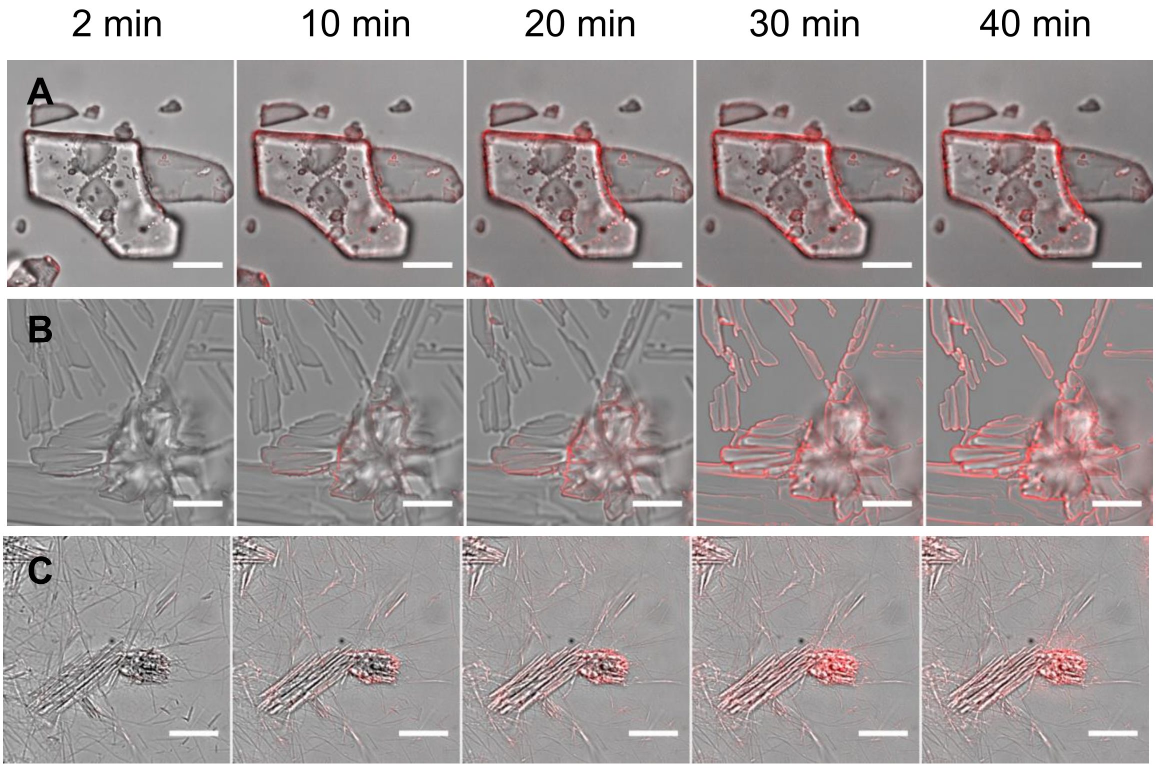

Supplementary Figure 7. Real-time fluorescence imaging of adsorption at drug crystal surfaces over 40 min following treatment with aqueous NP**1**-P_{188}. Red coloured fluorescence overlaid on DIC images from Figure 15. Real-time fluorescence imaging of adsorption at drug crystal surfaces at time points of 2, 10, 20, 30, and 40 min following treatment with aqueous NP**1**-P_{188}. **A**. Naproxen (Supplementary Movie 11); **B**: Ibuprofen (Supplementary Movie 12); **C**: Paclitaxel (Supplementary Movie 13). Scale bars = 20 μm.

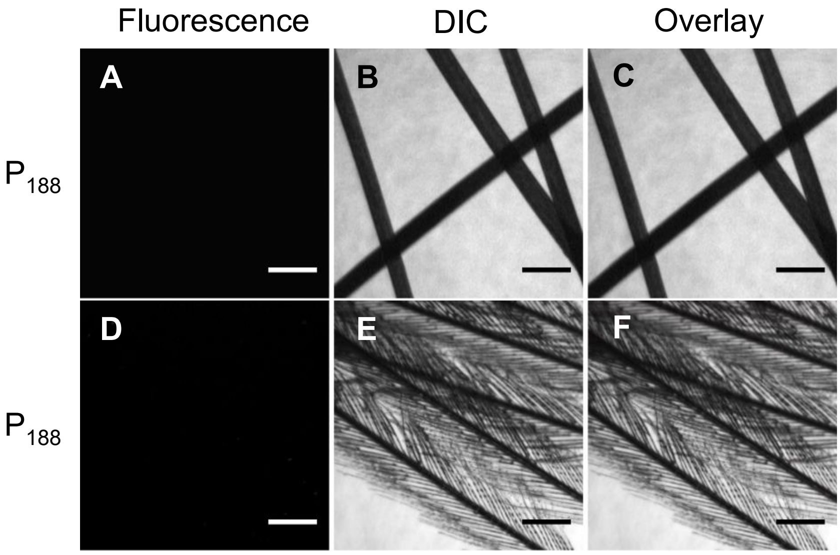

Supplementary Figure 8. Negative control experiments. Fluorescence and DIC images of hair (A-C) and feather fibres (D-F) following treatment with P_{188} (1.19 mM). Scale bar = 250 µm.

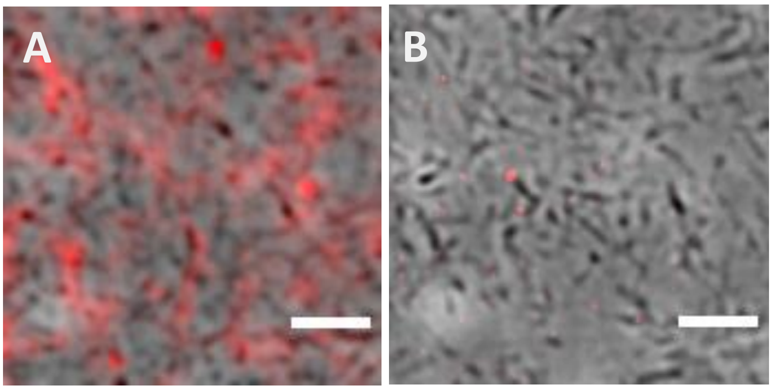

Supplementary Figure 9. Staining of insulin fibrils. Fluorescence and DIC overlay images of fibrils after treatment with NP**1**-P_{188} (A) and 1.19 mM P_{188} (B). Scale bar = 5 µm.

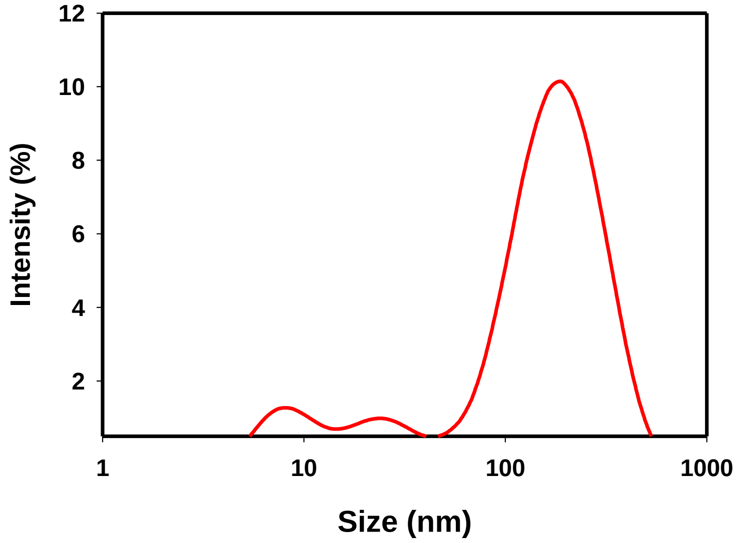

Sample	Size / nm	Pdi
NP**1**-P_{188}	205.56±0.61	0.61±0.01

Supplementary Figure 10. Particle sizing in biological media. DLS measurements for NP**1**-P_{188} (P_{188}**;** 1.19 mM, **1**; 5 µM) formed in DMEM.

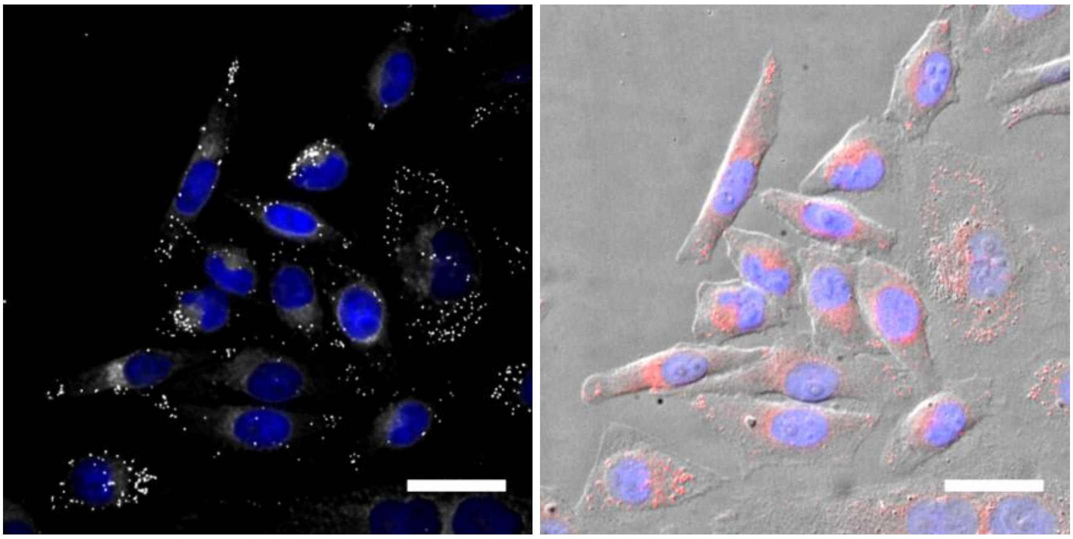

Supplementary Figure 11. HeLa Kyoto cell imaging of uptake of NP**1**-P_{188}. Additional independent experiment showing fixed images following 2 h incubation of HeLa Kyoto cells with NP**1**-P_{188}. Scale bar = 40 um.

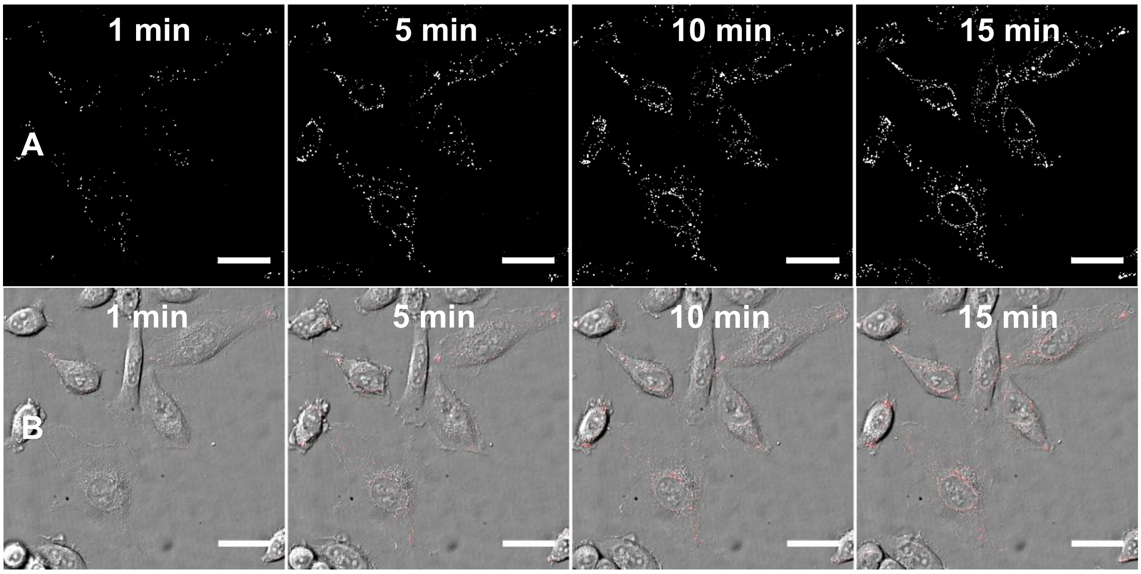

Supplementary Figure 12. Full field of view showing real-time live cell imaging of uptake, turning on and trafficking of NP**1**-P_{188} as described in Figure 20. See Supplementary Movie 17 and 18 for time-lapse video of A and B respectively. Scale bar = 40 um.

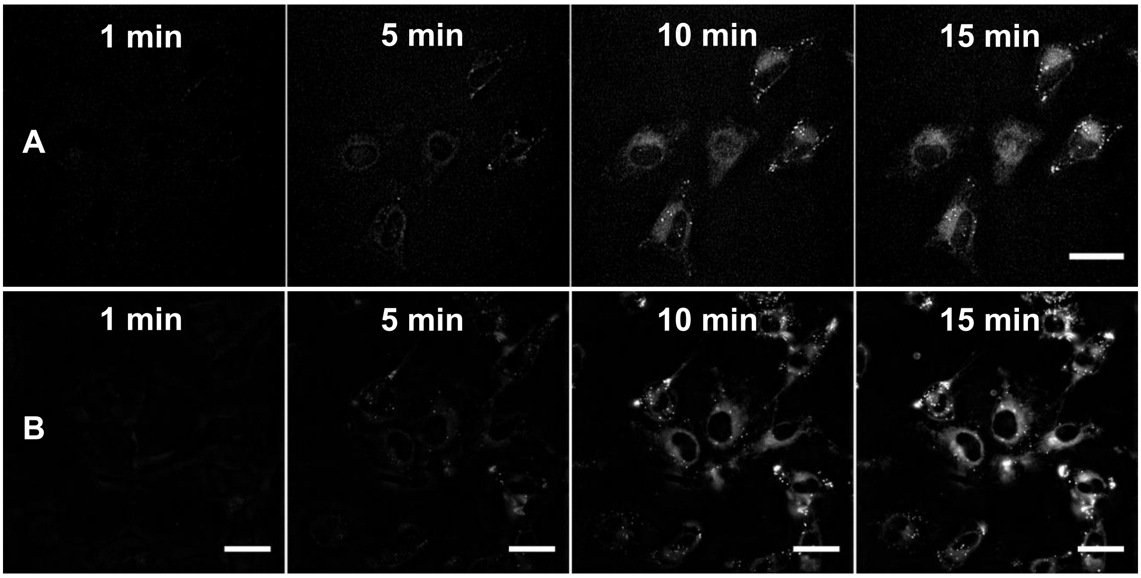

Supplementary Figure 13. Real-time live HeLa Kyoto cell imaging of NP**1**-P_{188}. Two additional independent experiments showing real-time live cell imaging of uptake, turning on and trafficking of NP**1**-$P_{188.}$ Scale bar = 40 um. See Supplementary Movies 19 and 20 for time-lapse video of experiment A and experiment B respectively.
