# Supplementary material for: Directed self-assembly of fluorescence responsive nanoparticles and their use for real-time surface and cellular imaging
Source: Nat Commun. 2017 Dec 1;8:1885. doi: 10.1038/s41467-017-02060-8 (PMC5709404; doi:10.1038/s41467-017-02060-8)
Supplement: Supplementary file 3 — Description of Additional Supplementary Files [file 41467_2017_2060_MOESM3_ESM.pdf]

Description of Additional Supplementary Files

File Name: Supplementary Movie 1
Description: Fluorescent naproxen crystals from 0-100 sec (of Fig. 6a)

File Name: Supplementary Movie 2
Description: Fluorescent ibuprofen crystals from 0-100 sec (of Fig. 6b).

File Name: Supplementary Movie 3
Description: Fluorescent paclitaxel crystals from 0-100 sec (of Fig 6c).

File Name: Supplementary Movie 4
Description: Fluorescent naproxen crystals from 0-100 sec with DIC overlay (of Supplementary Fig. 7a).

File Name: Supplementary Movie 5
Description: Fluorescent ibuprofen crystals 0-100 sec with DIC overlay (of Supplementary Fig. 7b).

File Name: Supplementary Movie 6
Description: Fluorescent paclitaxel crystals 0-100 sec with DIC overlay (of Supplementary Fig. 7c).

File Name: Supplementary Movie 7
Description: Fluorescent naproxen crystals 0-40 min (of Supplementary Fig. 9a).

File Name: Supplementary Movie 8
Description: Fluorescent ibuprofen crystals 0-40 min (of Supplementary Fig. 9b).

File Name: Supplementary Movie 9
Description: Fluorescent paclitaxel crystals 0-40 min (of Supplementary Fig. 9c).

File Name: Supplementary Movie 10
Description: Fluorescent naproxen crystals 0-40 min with DIC overlay (of Supplementary Fig. 10a).

File Name: Supplementary Movie 11
Description: Fluorescent ibuprofen crystals 0-40 min with DIC overlay (of Supplementary Fig. 10b).

File Name: Supplementary Movie 12
Description: Fluorescent paclitaxel crystals 0-40 min with DIC overlay (of Supplementary Fig. 10c).

File Name: Supplementary Movie 13
Description: Stability of adsorbed 1/P188 on naproxen crystal surface (of Fig. 6e).

File Name: Supplementary Movie 14
Description: Cellular delivery fluorescence turn-on (of Fig. 9a).

File Name: Supplementary Movie 15
Description: Cellular delivery fluorescence turn-on with DIC overlay (of Fig. 9b).

File Name: Supplementary Movie 16
Description: Intracellular vesicle tracking (of Fig. 9d).

File Name: Supplementary Movie 17
Description: Fluorescence images of the full field of view of Fig. 9a.

File Name: Supplementary Movie 18
Description: Fluorescence images of the full field of view of Fig. 9b with DIC overlay.

File Name: Supplementary Movie 19
Description: Fluorescence images of an additional independent cell delivery experiment.

File Name: Supplementary Movie 20
Description: Fluorescence images of an additional independent cell delivery experiment.
